# Editorial: Effects of performing arts training on the brain, (socio)cognitive and motor functions across the lifespan

**DOI:** 10.3389/fnhum.2023.1342325

**Published:** 2023-12-06

**Authors:** Leonie Kausel, Julia C. Basso, Noemí Grinspun, Claude Alain

**Affiliations:** ^1^Centro de Estudios en Neurociencia Humana y Neuropsicología (CENHN), Facultad de Psicología, Universidad Diego Portales, Santiago, Chile; ^2^Department of Human Nutrition, Foods, and Exercise, Virginia Tech, Blacksburg, VA, United States; ^3^School of Neuroscience, Virginia Tech, Blacksburg, VA, United States; ^4^Center for Health Behaviors Research, Fralin Biomedical Research Institute at Virginia Tech Carilion, Roanoke, VA, United States; ^5^Núcleo de Bienestar y Desarrollo Humano, Education Research Center (CIE-UMCE), Universidad Metropolitana de Ciencias de la Educación, Santiago, Chile; ^6^The School of Creative Arts Therapies, University of Haifa, Haifa, Israel; ^7^Institute of Medical Science, Temerty Faculty of Medicine, University of Toronto, Toronto, ON, Canada; ^8^Rotman Research Institute, Baycrest Health Sciences, Toronto, ON, Canada; ^9^Music and Health Research Collaboratory, Faculty of Music, University of Toronto, Toronto, ON, Canada; ^10^Department of Psychology, University of Toronto, Toronto, ON, Canada

**Keywords:** performing arts, brain, (socio)cognitive functions, motor functions, lifespan

Performing arts are a cultural expression that is ubiquitous around the world and consists of arts that are performed for an audience, such as music, dance, and drama. In recent years, there has been a growing interest in understanding how this expressive, and in essence social activity, impacts brain development and plasticity. This topic aimed to collect evidence on how the brain and (socio)cognitive and motor functions are influenced by performing arts training along the lifespan, deepening the current knowledge on this subject and helping to unravel the neurobiological mechanisms that underlie these changes. The five articles presented in this Research Topic explore research on an acting intervention, cover matters related to dance training, identify variables related to music sophistication, and focus on performing arts and musical training.

The first two articles are related to performing arts in older age. The first article by Rajesh et al. evaluates the impact of an acting intervention on brain activity of older adults during resting state functional magnetic resonance imaging (rs-fMRI). Prior research suggests that brain modularity, or brain regions that are distinctly and densely connected with other brain regions, decreases with age (Betzel et al., 2014), which may be related to outcomes of poorer executive functioning (Baniqued et al., 2017). In the article from Rajesh et al., older adults participated in an intervention two times a week for four weeks. The active intervention group enacted scenes with a partner, whereas the active control group learned about the history and styles of acting. The acting group showed an increase in brain modularity when compared to pre-intervention baselines and active controls. Also, performance on updating tasks was representative of the intervention group, but it was not possible to distinguish groups by means of evaluating interactions between post-intervention performance on updating and increases in brain modularity. Taken together, the results indicate that acting interventions in older age mitigate age effects on brain modularity activity and executive functions. The second article by Pentikäinen et al. summarizes the findings of a two year follow-up study on the impact of choir singing on aging cognition and wellbeing. In this study, older adult choir singers and demographically matched non-singers were assessed at three time points over two years on cognitive functioning and emotional and social wellbeing, using self-report questionnaires and standardized tests. Results showed that choir singers had higher verbal flexibility already at the first measuring time point that did not change over time, and that non-singers showed an enhancement in this task over measuring times. On the other hand, word knowledge, social inclusion and safety of the environment perception changed between singers and non-singers over time (enhancement in choir singers and decline in non-singers), even when there were no significant changes within groups. Importantly, shorter experience in choir singing was associated with greater improvement in word knowledge over the follow-up period. This indicates that verbal advantages acquired through singing are noticeable during the first training period. Both articles point toward results that indicate that performing arts could have a positive effect on older adults' brain and cognitive functions. This is especially important in light of the actual need for cost-effective interventions for the older population that could allow them to have a better life trajectory (WHO, 2017). As such, performing arts emerge as a promising approach that could help to achieve this goal.

The third article by Yang et al. explores how the experience of integrating the body and mind in dancers impacts the functional connectivity of the extended mirror neuron system (eMNS) during rs-fMRI. The eMNS is active during both action execution and observation (Ramsey et al., 2021) and could be modulated by dance training. Findings show that dancers have enhanced intra-regional functional connectivity within core eMNS areas (e.g., frontal gyrus, premotor cortex, basal ganglia). These results suggest that these mechanisms potentially could enable dancers to effortlessly integrate observed actions into their own motor skills by imitating and simulating dance movements.

The fourth article by Cui et al. deals with connectivity patterns measured during rs-fMRI related to music sophistication. Musical sophistication encompasses a broad spectrum of musical skills, such as singing abilities, and is not limited to individuals with formal music training (Müllensiefen et al., 2014). Interestingly, this study shows that only a small part of the connectivity patterns associated with music sophistication seem to relate to the effects of performing arts training beyond the effects of individual musical sophistication levels. In general their results emphasize the potential involvement of sensory regions in active engagement with music, the potential contribution of motor areas in processing emotions in music, and the potential impact of connectivity between the putamen and lingual gyrus in overall musical sophistication. Taken together, these findings suggest that there is still much to be understood on how connectivity patterns associated with constructs such as music sophistication arise and could be impacted by different forms of performing arts.

Finally, the fifth article by Korte et al. is a study that explores a dark side effect that sometimes accompanies performing arts mostly due to incorrect postures: chronic pain (Gasenzer et al., 2017; Swain et al., 2018). This study evaluated pain perception and pain catastrophizing, a critical pain-related behavior and emotional concept, in musicians and non-musicians through self-report questionnaires. Interestingly, they found that pain catastrophizing was significantly worse in non-musicians compared to musicians, even though the last ones that practiced in college (a study modality preferred by those students that aim to attain elite music status) reported perceiving pain for significantly longer. This evidence suggests that musicians do not seem to develop maladaptive pain management strategies, despite the fact that they can suffer from chronic pain.

In conclusion, the collection of articles published in this Research Topic contributes to our understanding of the complex interplay between performing arts and brain and (socio)cognitive and motor functions. The findings exposed here encourage further exploration of performing arts as a valuable and multifaceted model to study brain plasticity and as a tool for potential applications to promote wellbeing in older adults.

## Author contributions

LK: Writing – original draft, Writing – review & editing. JB: Writing – review & editing. NG: Writing – review & editing. CA: Writing – review & editing.

## References

[B1] Baniqued P. L. Gallen C. L. Voss M. W. Burzynska A. Z. Wong C. N. Cooke G. E.. (2017). Brain network modularity predicts exercise-related executive function gains in older adults. Front. Aging Neurosci. 9, 426. 10.3389/fnagi.2017.00426 29354050 PMC5758542

[B2] Betzel R. F. Byrge L. He Y. Goñi J. Zuo X.-N. Sporns O. (2014). Changes in structural and functional connectivity among resting-state networks across the human lifespan. NeuroImage 102, 345–357. 10.1016/j.neuroimage.2014.07.067 25109530

[B3] Gasenzer E. R. Klumpp M.-J. Pieper D. Neugebauer E. A. M. (2017). The prevalence of chronic pain in orchestra musicians. German Med. Sci. 15, Doc01. 10.3205/000242 28149258 PMC5238713

[B4] Müllensiefen D. Gingras B. Musil J. Stewart L. (2014). The musicality of non-musicians: an index for assessing musical sophistication in the general population. PLoS ONE 9, e89642. 10.1371/journal.pone.0089642 24586929 PMC3935919

[B5] Ramsey R. Kaplan D. M. Cross E. S. (2021). Watch and learn: the cognitive neuroscience of learning from others' actions. Trends Neurosci. 44, 478–491. 10.1016/j.tins.2021.01.007 33637286

[B6] Swain C. T. V. Bradshaw E. J. Whyte D. G. Ekegren C. L. (2018). The prevalence and impact of low back pain in pre-professional and professional dancers: a prospective study. Phys. Ther. Sport 30, 8–13. 10.1016/j.ptsp.2017.10.006 29257984

[B7] WHO (2017). Global Strategy and Action Plan on Ageing and Health. World Health Organization. Available online at: https://www.who.int/publications/i/item/9789241513500 (accessed November 19, 2023).

